# Respiratory mechanics and gas exchange in an ovine model of congenital heart disease with increased pulmonary blood flow and pressure

**DOI:** 10.3389/fphys.2023.1188824

**Published:** 2023-06-09

**Authors:** Joao Henrique N. Soares, Gary W. Raff, Jeffrey R. Fineman, Sanjeev A. Datar

**Affiliations:** ^1^ Department of Surgical and Radiological Sciences, School of Veterinary Medicine, University of California, Davis, Davis, CA, United States; ^2^ Department of Surgery, School of Medicine, University of California, Davis, Davis, CA, United States; ^3^ Cardiovascular Research Institute, University of California, San Francisco, San Francisco, CA, United States; ^4^ Department of Pediatrics, School of Medicine, University of California, San Francisco, San Francisco, CA, United States

**Keywords:** congenital heart disease, left-to-right shunt, respiratory mechanics, gas exchange, surgical large animal model

## Abstract

In a model of congenital heart disease (CHD), we evaluated if chronically increased pulmonary blood flow and pressure were associated with altered respiratory mechanics and gas exchange. Respiratory mechanics and gas exchange were evaluated in 6 shunt, 7 SHAM, and 7 control age-matched lambs. Lambs were anesthetized and mechanically ventilated for 15 min with tidal volume of 10 mL/kg, positive end-expiratory pressure of 5 cmH_2_O, and inspired oxygen fraction of 0.21. Respiratory system, lung and chest wall compliances (C_rs_, C_L_ and C_cw_, respectively) and resistances (R_rs_, R_L_ and R_cw_, respectively), and the profile of the elastic pressure-volume curve (%E_2_) were evaluated. Arterial blood gases and volumetric capnography variables were collected. Comparisons between groups were performed by one-way ANOVA followed by Tukey-Kramer test for normally distributed data and with Kruskal–Wallis test followed by Steel-Dwass test for non-normally distributed data. Average C_rs_ and C_L_ in shunt lambs were 30% and 58% lower than in control, and 56% and 68% lower than in SHAM lambs, respectively. C_cw_ was 52% and 47% higher and R_cw_ was 53% and 40% lower in shunt lambs compared to controls and SHAMs, respectively. No difference in %E_2_ was identified between groups. No difference in respiratory mechanics was observed between control and SHAM lambs. In shunt lambs, R_cw_, C_rs_ and C_L_ were decreased and C_cw_ was increased when compared to control and SHAM lambs. Pulmonary gas exchange did not seem to be impaired in shunt lambs when compared to controls and SHAMs.

## Introduction

Congenital heart disease (CHD) is the most common birth defect, affecting ∼8 in every 1000 live births in the United States (Hoffman and Kaplan, 2002) and the most common of these, ventricular septal defects and patent ductus arteriosus (VSDs, PDA) result in left to right shunting and increased pulmonary blood flow (PBF). Children with this physiology often live with increased PBF and pulmonary artery pressure (PAp) for weeks or months before their defects are surgically corrected (Mitchell et al., 1971), and the associated respiratory dysfunction and morbidity is well-described (Fitzgerald and Sherwood, 2007). Several studies evaluating respiratory mechanics in patients with CHD and increased PBF have consistently found that these patients have restrictive pulmonary function (Healy et al., 2012). For example, Howlett (Howlett, 1972) compared lung mechanics in infants with and without CHD, matched for length and height, and found that those with CHD had decreased lung compliance (C_L_). Similarly, Bancalari and colleagues (Bancalari et al., 1977), when comparing respiratory mechanics in infants with CHD and either increased or decreased PBF, found that respiratory rate and pulmonary resistance were higher in infants with increased PBF and that their C_L_ was significantly lower than in those with decreased PBF (Bancalari et al., 1977). In these patients, the decrease in C_L_ correlated with elevated mean pulmonary artery pressure. Collectively, these results suggest that the derangements in respiratory mechanics observed in CHD is caused by a combination on increase in PBF and PAp.

Many of these children continue to have abnormal respiratory mechanics several years after successful surgical correction of their cardiac defect. In a study from the mid-1990s, pulmonary function testing in children, who were on average 9 years out from a successful VSD repair, revealed that nearly three-quarters of them had decreased lung compliance and increased lung recoil pressure (Sulc et al., 1996). This respiratory dysfunction lasts into adulthood (Rex et al., 2019) and worsens with age (Maagaard et al., 2020). However, the underlying mechanisms that lead to this abnormal and persistent respiratory physiology are not at all understood. In order to better understand the cardiorespiratory interactions that might contribute to respiratory dysfunction in patients who experience chronically increased PBF, a large animal model is needed.

We have previously developed a surgical large animal model of increased PBF and pressure that we have used to study derangements of the pulmonary vasculature in this setting (Reddy et al., 1995; Black et al., 2000; Steinhorn et al., 2001; Black et al., 2003; Lakshminrusimha et al., 2007; Datar et al., 2012; Oishi et al., 2013; Datar et al., 2014; Johnson Kameny et al., 2019; Zhu et al., 2020; Boehme et al., 2021). In late gestation fetal lambs, a large 8 mm vascular graft is placed between the ascending aortic trunk and the main pulmonary artery (Reddy et al., 1995). After spontaneous delivery and with air-breathing, as pulmonary vascular resistance falls, there develops unrestricted left to right shunting that leads to chronic and torrential PBF (Reddy et al., 1995). Just as with infants and children with CHD and increased PBF (Healy et al., 2012), these ‘shunt’ lambs are tachypneic and have labored work of breathing. In addition, chest radiography and computed tomography demonstrate generalized cardiomegaly with increased vascular markings, arterial and venous distention, and patchy interstitial pulmonary infiltrates in shunt lambs (Datar et al., 2012). Thus, shunt lambs replicate with high fidelity the cardiorespiratory pathophysiology of unrepaired patients with CHD and increased PBF and pressure.

The aim of this study was to evaluate the respiratory mechanics and gas exchange in this large animal model of CHD, and to provide a benchmark for future studies that evaluate respiratory mechanics after repair of the cardiac defect (closure of the shunt).

## Materials and methods

### Animals

This study followed the ARRIVE guidelines and was approved by the Institutional Animal Care and Use Committee of the University of California, Davis (n^o^ 21594).

Twenty lambs aging between 28 and 46 days old and weighing between 10 and 21 kg were allocated in three experimental groups: control, shunt, and SHAM. The shunt group had six lambs with a surgically created anastomosis between the pulmonary artery and the aorta, as a model of CHD with increased PBF and pressure, previously described in detail elsewhere (Reddy et al., 1995; Steinhorn et al., 2001; Datar et al., 2012). In summary, after a lateral thoracotomy, an 8.0-mm Gore-tex vascular graft (W.L. Gore and Associates, Milpitas, CA) was anastomosed between the ascending aorta and main pulmonary artery in fetuses at 135–138 days of gestation (full term is 145 days) from mixed-breed Western pregnant ewes. The control group was composed of seven lambs that did not undergo any fetal surgical procedure but in two cases were twins of shunts. The SHAM group was designed to exclude any unanticipated effects that a fetal thoracotomy procedure might have on our assessment of respiratory mechanics. We performed sham-shunt operations on late-gestation fetal lambs, as above and (Reddy et al., 1995; Steinhorn et al., 2001; Datar et al., 2012). These SHAMs experienced identical anesthesia and instrumentation, including subcutaneous and intramuscular injections of local anesthetic at the thoracotomy site. An incision in the fourth intercostal space through the intercostal muscles was followed by full retraction of the ribs as was done when placing the actual aortopulmonary anastomosis. The deep and superficial planes of the intercostal incision site were subsequently suture-closed in a manner identical to the actual shunt procedure (Steinhorn et al., 2001; Datar et al., 2012).

### Anesthesia and instrumentation

Anesthesia was induced in lambs by the intravenous administration of propofol (4 mg/kg) and ketamine (2.5 mg/kg). A cuffed endotracheal tube between 5.0 and 7.0 internal diameter was orally placed into the trachea and connected to a rebreathing circle system. During the entire experiment, anesthesia was maintained by an end-tidal concentration of isoflurane (ET_ISO_) between 1.2% and 2.0% and the lambs were positioned in sternal recumbency. Mechanical ventilation was performed in volume control (constant flow) with a tidal volume (V_T_) of 10 mL/kg, positive end-expiratory pressure (PEEP) of 5 cmH_2_O and target inspiratory fraction of oxygen (FIO_2_) of 0.21 (Flow-I C-20, MAQUET Medical Systems United States of America, Wayne, NJ). Respiratory rate (RR) was adjusted to maintain the end-expiratory partial pressure of CO_2_ (P_ET_CO_2_) between 35 and 45 mmHg. Heart rate and rhythm were monitored with electrocardiogram, and pulse oximetry (SpO_2_) was measured at the tongue, tail, or ear (AS/3, Datex/Ohmeda, Finland). A 22-gauge catheter (Surflo, Terumo Corporation, NJ, United States of America) was placed in the auricular or coccygeal artery for the measurement of blood pressure and sampling to measure blood gases. The blood pressure transducer was maintained at the level of the heart and was calibrated against a mercury column prior to the experiments. A pediatric mainstream flow/CO_2_ sensor (Respironics Novametrix, LLC, Wallingford, CT) was positioned between the endotracheal tube and the breathing system to measure P_ET_CO_2_ and to collect the continuous signals of CO_2_ partial pressure (PCO_2_) for an offline volumetric capnography analysis (NM3, Philips Respironics, Wallingford, CT). A screen pneumotachometer (4500, Hans Rudolph Inc., Shawnee, KS) connected to a differential pressure transducer (DPL 2.5, Hugo Sacks Elektronik-Harvard Apparatus, Germany) was positioned distal to the flow/CO_2_ sensor and used to measure airflow (V̇). Airway opening pressure (P_ao_) was measured from a port located on the pneumotachometer by a calibrated pressure transducer (MPX, Hugo Sacks Elektronik-Harvard Apparatus, Germany). A polypropylene balloon tipped catheter was positioned in the esophagus of the lambs for the measurement of esophageal pressure (P_eso_) as an estimate of pleural pressure. The esophageal catheter was connected to a pressure transducer (P75, Hugo Sacks Elektronik-Harvard Apparatus, Germany) and its correct position verified by the observation of equal variation in P_ao_ and P_eso_ during the external compression of the thorax while the endotracheal tube was occluded, as described by Lanteri and coworkers (Lanteri et al., 1994). Details of the esophageal balloon catheter used in this study and a graphical representation of the dynamic response of the P_eso_ and P_ao_ during the occlusion maneuver is presented in the Supplementary Figure S1. Rectal temperature was monitored during the experiments and reported in Celsius degrees.

### Experimental protocol

**FIGURE 1:**
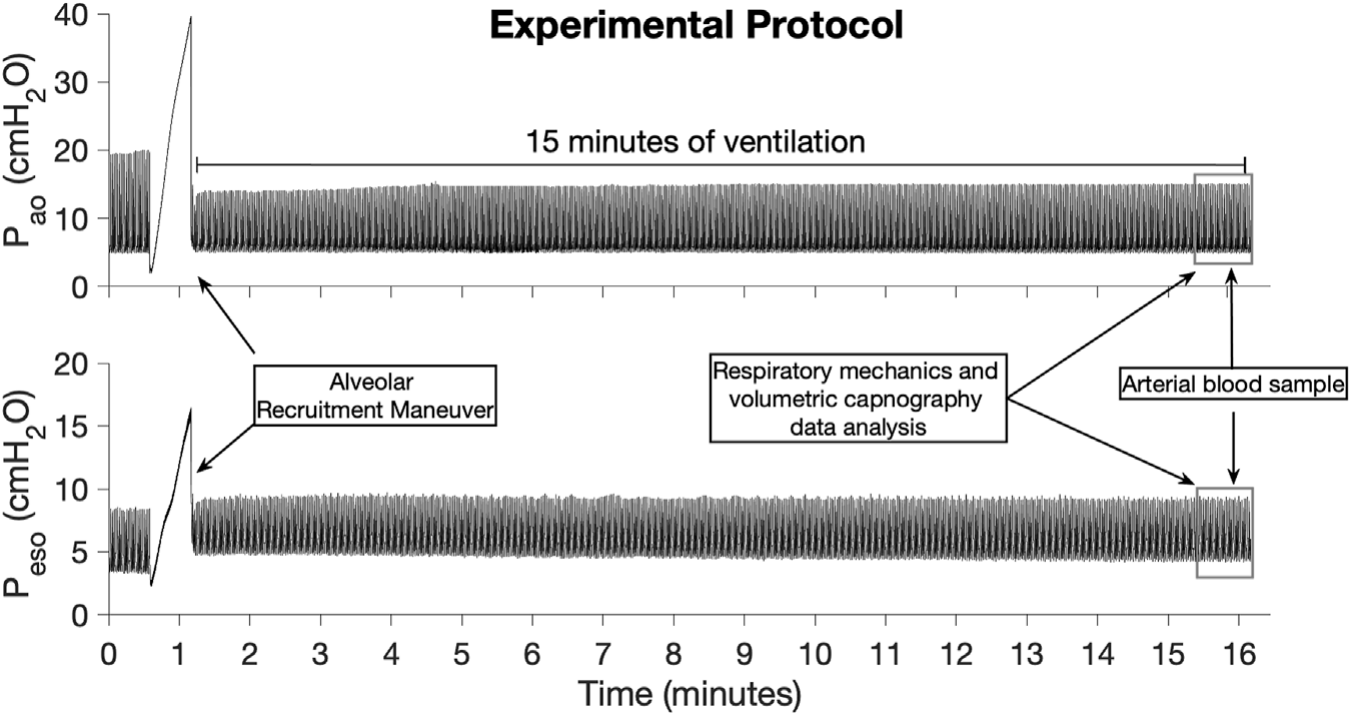
Timeline of the experiments illustrated by the airway opening pressure (P_ao_) and esophageal pressure (P_eso_) signals collected in one of the control lambs.

After instrumentation, an alveolar recruitment maneuver based on a slow inflation of the lungs with 2–3 L/min until P_ao_ reached 40 cmH_2_O was performed to standardize the lung volume history in all lambs. After that, the lambs were ventilated for 15 min with the same ventilatory settings used during the instrumentation. During the last minute of ventilation, an arterial blood sample of 1 mL was collected in heparinized syringes (PICO 70, Radiometer America Inc., IL), for the immediate measurement of the arterial partial pressure of O_2_ (PaO_2_) and CO_2_ (PaCO_2_) (ABL 825, Radiometer America Inc., Brea, CA). Arterial blood gas values were not corrected for body temperature because no algorithm for temperature correction was found for sheep blood. Dynamic respiratory mechanics and volumetric capnography data were collected during the last 2 minutes of ventilation. After the 15 min of ventilation protocol, the delivery of isoflurane was stopped, and the lambs recovered from anesthesia. Once the lambs were extubated and presented coordinated movements, they were placed in a transport crate and returned to the animal vivarium facility. The timeline of the experiment is presented in Figure 1.

### Data acquisition and calibrations

The analog signals of arterial blood pressure, V̇, P_ao_, and P_eso_ were passed through signal conditioner modules (TAM-A Modified, Hugo Sacks Elektronik–Harvard Apparatus GmbH, Germany) and the analog signal of PCO_2_ and a second V̇ signal were collected from the analog output port of the volumetric capnography monitor (NM3, Philips Respironics, Wallingford, CT) to compute physiologic dead space (V_Dphys_) and anatomic dead space (V_DANAT_). All analog signals were digitized at 400 Hz using an eight channel, 14-bit analog to digital board (NI USB 6009, NI, Austin, TX). The digital signals were acquired by a dedicated data acquisition system (LabView 2017; NI, Austin, TX) and saved on a personal computer for offline processing.

Calibration curves for the P_ao_ and P_eso_ transducers were estimated by a simple linear regression function of 5 pressure points (0, 10, 20, 30 and 40 cmH_2_O) obtained with a water column and their respective voltage recorded on the data acquisition system. Calibration of the flow measurements was made by a polynomial curve fitting procedure (Giannella-Neto et al., 1992) applied to a wide range of flows generated by a volumetric calibration syringe (5540, Hans Rudolph Inc., Shawnee, KS). The gas mixture used to calibrate the pneumotachometer had a composition similar to that used during the experiments (O_2_ and isoflurane concentrations of 21% and 1.5%, respectively). The infrared CO_2_ sensor was calibrated by simple linear regression using standard calibration tanks containing 3.1, 5.0, 8.1 (Puritan Bennet, Overland Park, KS) and 11.2% CO_2_ (Radiometer America Inc., Brea, CA). Barometric pressure (P_B_) was measured by a digital barometer (Fisher Scientific, Hampton, NH) at the time of calibration. All calibrations were performed immediately before each experiment.

### Gas exchange

Alveolar partial pressure of oxygen (PAO_2_) was calculated using the alveolar air equation described below using partial pressure of gases at 37°C.

$${PAO}_{2}={FIO}_{2}\times \left(P_{B}\;–\;47\right)\;–\;{PaCO}_{2}/R\times \left[1\;–\;{FIO}_{2}\times \left(1\;–\;R\right)\right]$$ (1)

where P_B_ is the barometric pressure measured in mmHg at the time of the experiment, R was the respiratory quotient considered 0.74 for sheep (Keenan et al., 1990). PAO_2_—PaO_2_ (P_A-a_O_2_) was calculated for each animal.

A previously validated 4-parameter model was fitted to the volumetric capnography signals using a custom-made implementation of Gauss-Newton algorithm (Motta-Ribeiro et al., 2020).

$${PCO}_{2}\left(V\right)=\frac{aV+b}{1+e^{d\left(c-V\right)}}$$ (2)

where V is volume, PCO_2_ is the partial pressure of CO_2_ in mmHg and a, b, c and d are parameters of the model.

V_Danat_ and physiologic dead space fraction (V_Dphys_/V_T_) were calculated as follows. V_Danat_ was considered as the inflexion point of the 4-parameter model (parameter c) (Motta-Ribeiro et al., 2020), and was also expressed as a fraction of V_T_ (V_Danat_/V_T_).

$$V_{Dphys}/V_{T}=\left({PACO}_{2}\;–\;PĒ{CO}_{2}\right)/{PACO}_{2}$$ (3)

where PACO_2_ is the mean alveolar CO_2_ partial pressure (mmHg) calculated from the middle part of the phase III of the volumetric capnography curve as described by Tusman et al. (Tusman et al., 2011), and PĒCO_2_ is the mixed expired CO_2_ partial pressure. Alveolar dead space fraction (V_Dalv_/V_T_) was calculated as V_Dphys_/V_T_–V_Danat_/V_T_. The slope of the phase III (SIII) was considered the asymptotic slope of the model (parameter a) and was normalized (SIII_n_) for the mixed expired fraction of CO_2_ (FĒCO_2_), calculated by the area under the curve of the volumetric capnogram divided by V_T_.

### Respiratory mechanics

Volume (V) was calculated by the numeric integration of the V̇ signal and the values of V̇ and V were reported as BTPS. The endotracheal resistive pressure (P_ResETT_) was calculated using a quadratic volume-dependent model as:

$$P_{ResETT}\;\left(t\right)=\left(K_{1ETT}+K_{2ETT}\times \left|\dot{V}\right|\left(t\right)\right)\times \dot{V}\left(t\right)+{In}_{ETT}\times \dot{\dot{V}}$$ (4)

where t is time, V̇̇ was the acceleration of the gases, and K_1ETT_, K_2ETT_ and In_ETT_ were the endotracheal tube values of linear resistance, flow-dependent resistance and inertance, respectively, obtained from reported values of ETT sizes 5.0 to 6.5 (Guttmann et al., 2000). To exclude the resistive properties of the ETT on the estimates of respiratory system and lung mechanics, tracheal pressure (P_Tracheal_) was calculated for the entire respiratory cycle by P_ao_ (t)—P_ResETT_ (t).

Dynamic respiratory mechanics was evaluated using a single-compartment model including a volume-dependent elastance and an inertial term. Respiratory system, lung and chest wall respective volume-independent elastance (E_1rs_, E_1L_, and E_1cw_), volume-dependent elastance (E_2rs_, E_2L_, and E_2cw_), resistance (R_rs_, R_L_, and R_cw_) and inertance (In_rs_, In_L_, and In_cw_) were estimated by multiple linear regression (least square method) of the models of the equation of motion using their respective driving pressures (Lanteri et al., 1995a).

$$P_{Tracheal}\;\left(t\right)={In}_{rs}\times \dot{\dot{V}}+R_{rs}\times \dot{V}\left(t\right)+\left(E_{1rs}+E_{2rs}\times V\left(t\right)\right)\times +P_{Tracheal,0}$$ (5)

$$P_{tp}\left(t\right)={In}_{L}\times \dot{\dot{V}}+R_{L}\times \dot{V}\left(t\right)+\left(E_{1L}+E_{2L}\times V\left(t\right)\right)\times V\left(t\right)+P_{tp,0}$$ (6)

$$P_{eso}\left(t\right)={In}_{cw}\times \dot{\dot{V}}+R_{cw}\times \dot{V}\left(t\right)+\left(E_{1cw}+E_{2cw}\times V\left(t\right)\right)\times V\left(t\right)+P_{eso,0}$$ (7)

where P_tp_ is transpulmonary pressure (P_Tracheal_–P_eso_), and P_Tracheal,0_, P_tp,0_, and P_eso,0_ are P_Tracheal_, P_tp_ and P_eso_, respectively, when V, $\dot{V}$ and $\dot{\dot{V}}$ are zero. An inertial term (In × $\dot{\dot{V}}$ ) was included in the models to improve the accuracy of elastance and resistance estimates since a relatively high *f*_R_ (20–40 breaths/minute) was used during the experiments (Lanteri et al., 1999). All estimates of elastances, resistances and inertances were reported as their average during the last 30 cycles of ventilation before the arterial blood sampling. Total elastance of respiratory system (E_rs_), lung (E_L_) and chest wall (E_cw_) were calculated by the sum of their respective volume-dependent and independent terms (total elastance = E_1_ + E_2_ × V_T_). An index of tidal alveolar overdistention and recruitment/derecruitment (%E_2_) was calculated according to Eq. (8) (Carvalho et al., 2008; Beda et al., 2017):

$$\%E_{2}=100\times \frac{\left(E_{2}\times V_{T}\right)}{\left(E_{1}+\left|E_{2}\right|\times V_{T}\right)}$$ (8)

If the elastic pressure-volume curve has an upward concavity, %E_2_ values are positive indicating that E_rs_ increases during inspiration. On the other hand, a negative %E_2_ reveals a downward concavity on the elastic pressure-volume curve due to a decrease in E_rs_ during inspiration. If the relation between elastic pressure and volume is linear %E_2_ approaches 0%. Values of %E_2_ higher than 20% and lower than −10% have been associated to tidal overdistention and recruitment/derecruitment, respectively, with values between −10% and 20% considered linear distention (Beda et al., 2017). Compliance was calculated for the total respiratory system (C_rs_), chest wall (C_cw_) and lungs (C_L_) by 1/E_rs_, 1/E_cw_ and 1/E_L_, respectively, and normalized to the body weight in kg of each lamb. R_rs_, R_L_ and R_cw_ were normalized to the body weight of each lamb using a power law described by Stahl (Stahl, 1967).

### Other measurements

From the same arterial blood sample used for the P_a_O_2_ and P_a_CO_2_ measurements, pH was measured and bicarbonate ion (HCO_3_^−^) concentration calculated. In addition, the arterial blood concentrations of lactate, glucose, Na^+^, Cl^−^, K^+^ and Ca^++^ were measured. All those measurements were performed by the same analyzer (ABL 825, Radiometer America Inc., Brea, CA).

### Statistical analysis

The main variables of this study were C_rs_, C_L_, C_cw_, R_rs_, R_L_, and R_cw_. Secondary variables were P_A-a_O_2_, SIII_n_, V_Dbohr_/V_T_, V_Danat_ and V_Dalv_/V_T_. Shapiro-Wilk test was used to verify normality of the continuous variables. Normally distributed data were reported as mean ±95% confidence interval and non-normally distributed data as median (range). Comparisons between groups with normal and non-normal distribution were performed by one-way analysis of variance followed by a Tukey-Kramer HSD test and a Kruskal–Wallis test followed by Steel-Dwass test, respectively. Statistical analysis was performed with a commercial statistics software (JMP Pro16.0.0, SAS Institute Inc., NC, United States of America) and *p* < 0.05 was considered enough to reject the null hypothesis.

## Results

All 20 lambs successfully completed the experiments. Arterial blood gas data from one lamb in the control group were not included in the results because of a malfunction in the blood gas analyzer on the day of the experiment. Because the esophageal balloon catheter got displaced during the experiment of one SHAM lamb, data of C_L_, C_cw_, R_L_ and R_cw_ of only 6 SHAM lambs are presented.

The demographic data and rectal temperature from shunt, control and SHAM lambs are presented in Table 1. There was no difference of age, body weight and rectal temperature between groups. Ventilatory variables and ET_ISO_ measured during the experiments are presented in Table 2. As expected, there was no significant difference between groups for V_T_, PEEP, ET_ISO_, and FIO_2_ between groups. In addition, *f*_R_ and peak tracheal pressure were not different between groups.

**TABLE 1:** Demographic data and rectal temperature in shunt, control and SHAM lambs mechanically ventilated for 15 min (mean ±95% confidence interval).

	Age (days)	BW (kg)	Temp (^o^C)
Shunt (n = 6)	33 ± 4	11.2 ± 3.0	39.5 ± 0.6
Control (n = 7	37 ± 4	14.3 ± 2.7	39.6 ± 0.6
SHAM (n = 7)	36 ± 4	14.5 ± 2.7	39.8 ± 0.5

BW, body weight; V_T_, tidal volume; *f*_R_ = respiratory rate; bpm = breaths/minute; PEEP, positive end-expiratory pressure; P_Trachealpeak_ = peak tracheal airway pressure; ET_ISO_, end-tidal concentration of isoflurane; FIO_2_ = inspired fraction of O_2_; and Temp = rectal temperature.

**TABLE 2:** Ventilatory variables and isoflurane end-tidal concentration (ET_ISO_) in shunt, control and SHAM lambs mechanically ventilated for 15 min [values are expressed as mean ±95% confidence interval or median (range)].

	V_T_ (mL/kg)	PEEP (cmH_2_O)	P_Trachealpeak_ (cmH_2_O)	ET_ISO_ (%)	FIO_2_
Shunt (n = 6)	9.8 ± 0.4	5 ± 0	17 ± 2	1.6 ± 0.2	0.21 (0.21 0.22)
Control (n = 7)	9.7 ± 0.4	5 ± 0	14 ± 2	1.5 ± 0.2	0.22 (0.21 0.23)
SHAM (n = 7)	10.0 ± 0.4	5 ± 0	15 ± 2	1.6 ± 0.2	0.22 (0.21 0.23)

**FIGURE 2:**
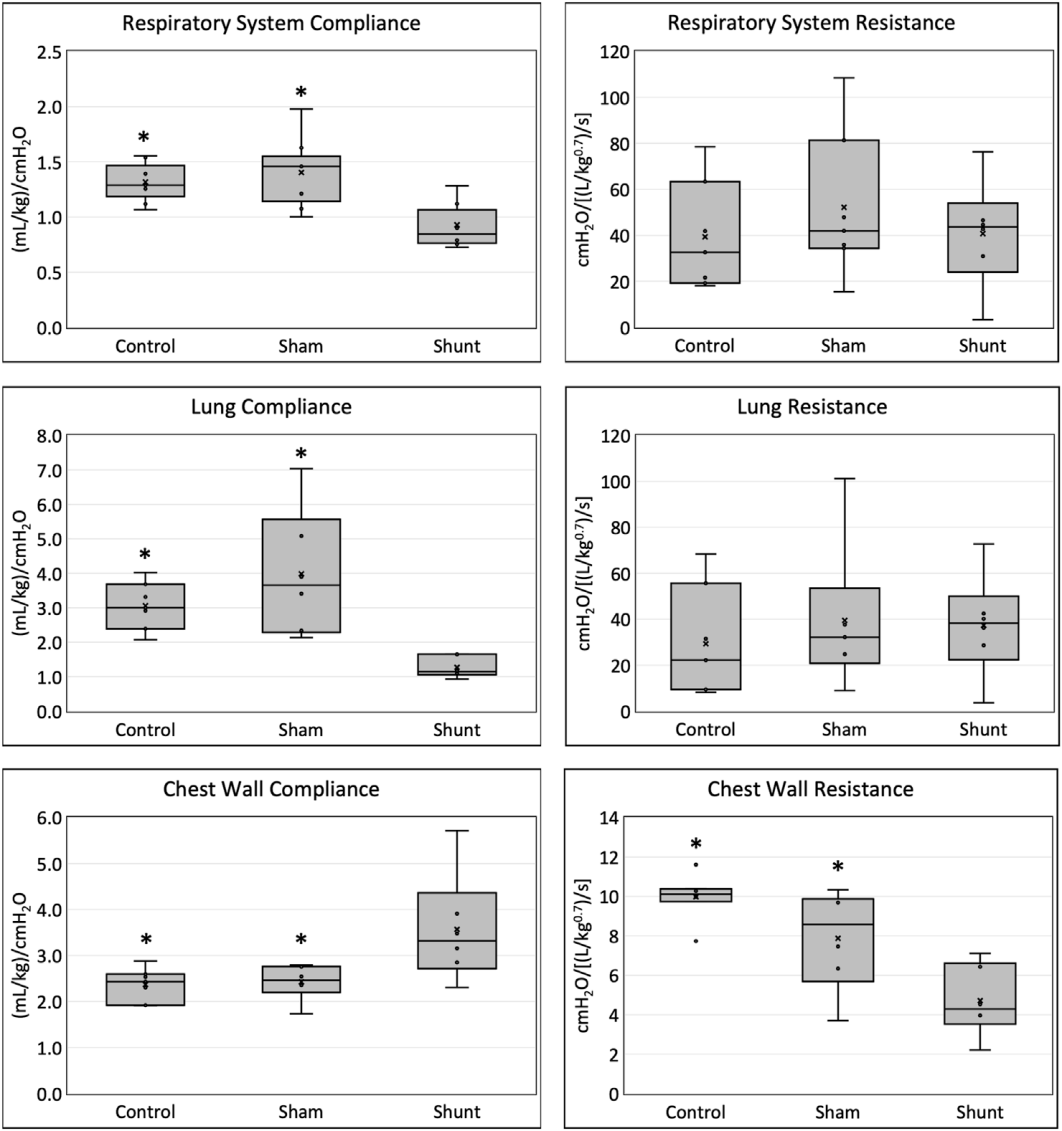
Respiratory system, lung and chest wall compliances and resistances in shunt, control and SHAM lambs mechanically ventilated for 15 min (* = significant difference between shunt lambs).

The main respiratory mechanics results of control, SHAM, and shunt lambs are reported in Figure 2. Average C_rs_ and C_L_ in shunt lambs were 30% and 58% lower than in control, and 56% and 68% lower than in SHAM lambs, respectively. C_cw_ was 52% and 47% higher and R_cw_ was 53% and 40% lower in shunt lambs compared to controls and SHAMs, respectively. No significant differences between groups were found for R_rs_ and R_L_. Median and range of %E_2_ were 10.8 (−11.3 21.3), −1.2 (−9.6 40.9) and 11.0 (7.1 32.8) in control, SHAM and shunt lambs, respectively, with no significant difference between groups. Based on the %E_2_ results, alveolar tidal recruitment/derecruitment was identified in two lambs of the control group (−11.3% and −10.2%%), and overdistention values were found in one lamb of each group (21.3% - control; 40.9% - SHAM, 32.8% - shunt). All parameters of the respiratory mechanics models used in this study are presented in Supplementary Table S2.

**FIGURE 3:**
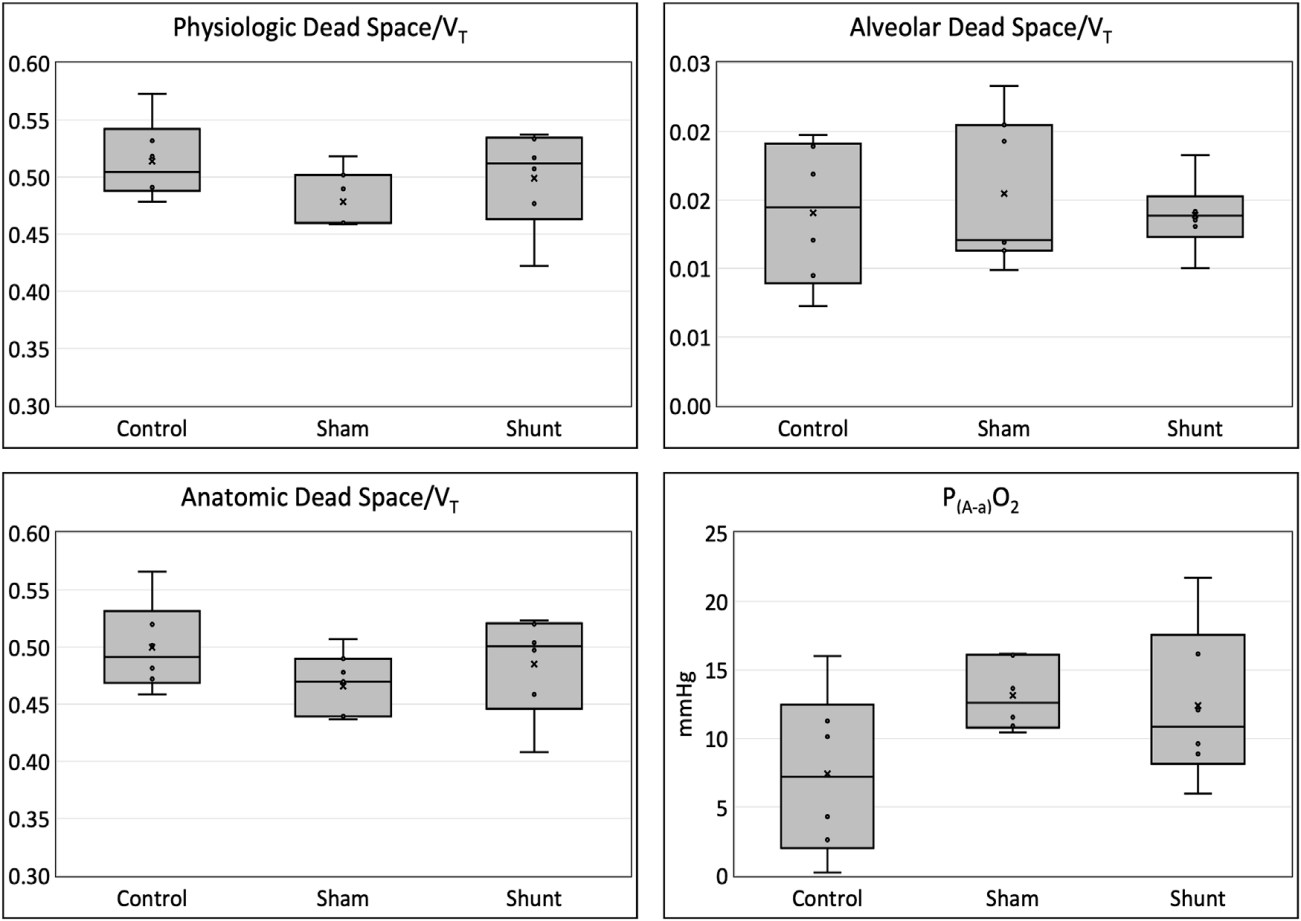
Physiologic, Anatomic and alveolar dead space fractions and alveolar-to-arterial oxygen partial pressure difference [P_(A-a)_O_2_] in shunt, control and SHAM lambs mechanically ventilated for 15 min.

Pulmonary gas exchange variables achieved during the experiments are reported in Table 3 and Figure 3. No significant difference between groups was identified for all pulmonary gas exchange variable. The arterial blood pH, and the concentrations of HCO_3_^−^, glucose, lactate, Cl^−^, K^+^, and Ca^++^ were also not different between groups (Table 4).

**TABLE 3:** Pulmonary gas exchange variables in control, SHAM and shunt lambs mechanically ventilated for 15 min. Values are expressed as mean ±95% confidence interval or median (range).

	P_a_CO_2_ (mmHg)	P_a_O_2_ (mmHg)	V_Danat_ (mL/kg)	SIII_n_
Control (n = 7)	42.2 ± 5.7†	99 ± 12†	4.9 ± 0.3	1.60 (0.93 3.08)
SHAM (n = 7)	37.5 ± 5.3	96 ± 12	4.6 ± 0.3	1.38 (1.23 1.95)
Shunt (n = 6)	46.7 ± 5.7	85 ± 12	4.6 ± 0.3	1.77 (0.20 2.52)

^a^ = significant difference; *p* < 0.05. † = data from only 6 individuals is presented. P_a_CO_2_ = arterial partial pressure of CO_2_; P_a_O_2_ = arterial partial pressure of O_2_; V_Danat_ = anatomic dead space; and SIII, is the slope of phase III, of the volumetric capnogram.

**TABLE 4:** Acid-base variables, lactate, glucose and electrolytes measured in the arterial blood of control and shunt lambs mechanically ventilated for 15 min. Values are expressed as mean ±95% confidence interval or median (range).

	pH	HCO_3_^−^ (mmol/L)	Lactate (mmol/L)	Glucose (mg/dL)	Na^+^ (mmol/L)	K^+^ (mmol/L)	Cl^−^ (mmol/L)	Ca^++^ (mmol/L)
Control (n = 6)	7.42 ± 0.06	26.0 ± 2.3	1.1 (0.4 1.8)	96 ± 13	143 ± 2	4.2 ± 0.4	110 ± 4	1.31 ± 0.04
SHAM (n = 7)	7.41 ± 0.05	22.8 ± 2.0	1.1 (0.8 2.3)	97 ± 12	145 ± 2	4.2 ± 0.3	114 ± 3	1.33 ± 0.05
Shunt (n = 6)	7.36 ± 0.04	24.3 ± 2.1	3.6 (0.9 5.9)	77 ± 13	144 ± 2	4.2 ± 0.4	111 ± 3	1.28 ± 0.04

## Discussion

The main findings of this study of respiratory mechanics and gas exchange in our surgical ovine model of CHD with increased PBF and pressure were: 1) C_rs_ and C_L_ were lower and C_cw_ was higher in shunt lambs than in age-matched control and SHAM lambs; 2) R_cw_ was lower in shunts, but R_rs_ and R_L_ were no different than in controls and SHAMs; and 3) pulmonary gas exchange was not different among control, SHAM and shunt lambs.

The ovine model of CHD with increased PBF and pressure used in this study replicates the typical features of the human disease, including elevated postnatal pulmonary arterial pressure and blood flow, with the associated derangements of pulmonary vascular structure and function (Reddy et al., 1995; Datar et al., 2012; Datar et al., 2014; Morris et al., 2018; Johnson Kameny et al., 2019; Zhu et al., 2020; Boehme et al., 2021). Although pulmonary artery pressure and pulmonary to systemic blood flow ratio (Q̇_p_/Q̇_s_) were not measured during these experiments, hemodynamics were measured in 5 shunt lambs during subsequent evaluations at between 8 and 15 weeks of age while under similar anesthetic conditions, which confirmed that these shunt lambs did experience increased PBF and elevated pulmonary artery pressure (Supplementary Table S4). This model has been used in prior investigations that examined the effects of CHD with increased PBF on pulmonary vascular remodeling (Reddy et al., 1995; Black et al., 2000; Steinhorn et al., 2001; Black et al., 2003; Lakshminrusimha et al., 2007; Oishi et al., 2008; Johnson Kameny et al., 2019), including the pulmonary lymphatic system (Datar et al., 2012; Datar et al., 2014; Datar et al., 2016; Boehme et al., 2021). However, an evaluation of the respiratory mechanics and gas exchange of this model have not been described in detail previously. Consequently, the findings of this study can be used to better understand the effects of CHD-driven increased PBF and pressure on pulmonary function: 1) as baseline measurements for future studies investigating the underlying mechanisms that lead to persistently abnormal respiratory mechanics after the cardiac defect has been repaired (Sulc et al., 1996; Abassi et al., 2019; Rex et al., 2019; Maagaard et al., 2020), and 2) how preservation of pulmonary vascular function in this setting, might improve variables of respiratory function.

### Respiratory mechanics

In this ovine model of CHD with increased PBF, C_rs_ was lower than in control and SHAM animals as a result of a lower C_L_. Similar results have been also reported in infants and children with CHD with increased PBF (Wallgren et al., 1960; Davies et al., 1962; Howlett, 1972; Bancalari et al., 1977; De Troyer and Englert, 1977; Baraldi et al., 1993; Lanteri et al., 1995b; Yau et al., 1996). The reasons for a lower C_L_ and C_rs_ in shunt lambs is likely related to the simultaneous increase in the pulmonary blood volume and pulmonary artery pressure (PAp) (Davies et al., 1962; Griffin et al., 1972; Howlett, 1972; Bancalari et al., 1977). Any discrepancies among these studies may be related to the variable degree of increased PBF and PAp observed in different studies, as suggested by Lanteri et al. (Lanteri et al., 1995b), and/or the different techniques used to evaluate respiratory mechanics used in each study. It has also been suggested that the pulmonary vascular remodeling in response to the chronic increase in PBF and PAp might create a stiffer pulmonary scaffold that contributes to a decreased C_L_ and C_rs_ as well (Davies et al., 1962). Indeed, shunt lambs have a higher density of pulmonary artery branches with thicker walls and an increased smooth muscle layer, particularly in the peripheral intra-acinar arteries (Reddy et al., 1995), which supports this mechanism for the decreased C_L_ and C_rs_ in the shunt lambs that we identified in this study. It is also possible that the increased PBF observed in these shunt lambs could lead to increased left atrial pressure resulting pulmonary venous congestion that also contributes to decreased C_L_. However, shunt lambs do not demonstrate a clinically relevant elevation in left atrial pressure, and this is not a model of left heart failure (Datar et al., 2012; Datar et al., 2014; Morris et al., 2018; Johnson Kameny et al., 2019). Lastly, Wallgren et al. (Wallgren et al., 1960) proposed that the decrease in C_L_ observed in infants with CHD and increased PBF could be related to compression atelectasis caused by their enlarged heart. This could certainly contribute to the decreased C_rs_ and C_L_ observed in shunt lambs because impressive right ventricular hypertrophy is a typical phenotypic trait of this model of CHD (Reddy et al., 1995; Datar et al., 2012).

The magnitude of the decrease in C_rs_ in the shunt lambs was less than the decrease in their C_L_ because their C_cw_ was higher than in the control lambs, which counterbalanced the effect of the lower C_L_ upon C_rs_. The increased C_cw_ observed in the shunt lambs could be related to the decreased elastic properties of a less muscular, less fatty chest wall, a result of the negative energy balance normally present in CHD with increased PBF (Menon and Poskitt, 1985). Moreover, the possible larger lung volume generated in the control and SHAM when compared to shunt lambs by the use of PEEP could have placed the chest wall in a lower compliance position of its pressure-volume curve. Our results identify an important limitation of studies that evaluate only C_rs_ to assess the degree of respiratory mechanics dysfunction in the individuals with CHD and PBF. Freezer and colleagues (Freezer et al., 1993) suggested that their inability to show decreased compliance in infants with CHD and increased PBF was possibly related to measuring C_rs_ and not C_L_ because the former included the contribution of the chest wall. Moreover, the accurate evaluation of C_L_ in individuals with CHD could have pivotal importance as a prognostic marker of improvement in lung function after surgical or pharmacological shunt closure: infant patients with lower preoperative C_L_ had the best improvement in lung function after surgical ligation of their PDA (Gerhardt and Bancalari, 1980).

The previously reported effects of CHD with increased PBF on R_rs_ and R_L_ are controversial, with some studies finding no difference (Howlett, 1972; Baraldi et al., 1993) while others an increase in R_rs_ or R_L_ (Bancalari et al., 1977; Freezer et al., 1993; Lanteri et al., 1995b; Yau et al., 1996). As discussed for the elastic properties, the differences among study results are likely because of differences in the methodologies used to evaluate R_rs_ and R_L_, and/or different degrees of pulmonary hemodynamic derangements in their patient population. Increases in R_rs_ or R_L_ previously reported in infants or children with CHD and increased PBF (Bancalari et al., 1977; Freezer et al., 1993; Lanteri et al., 1995b; Yau et al., 1996) have been attributed to a possible decreased airway diameter from compression by the enlarged heart and vascularly engorged lung structures, or from peribronchial fluid accumulation in the small airways (Hordof et al., 1977). Using a two-compartment model, Freezer and colleagues (Freezer et al., 1993) identified that the two components of R_rs_ (airway resistance and viscoelastic properties of lungs and chest wall) are affected in infants with CHD with high PBF. In addition, marked increases in R_rs_ were observed mainly in infants with Q̇_p_/Q̇_s_ greater than three. Unfortunately, Q̇_p_/Q̇_s_ was not calculated coincident with these pulmonary function experiments, but Q̇_p_/Q̇_s_ was subsequently calculated in five of the shunt lambs (at between 8 and 15 weeks of age); this revealed a mean Q̇_p_/Q̇_s_ of 2.3 (Supplementary Table S4). Even though Q̇_p_/Q̇_s_ higher than 3.0 is not uncommon in this model (Reddy et al., 1995; Datar et al., 2014), it is possible that the shunt lambs included in the study did not have enough increase in PBF to generate augmented R_rs_ and R_L_. Yet another possible reason that we did not observe a difference in R_rs_ and R_L_ between control and shunt lambs as previous studies (Bancalari et al., 1977; Freezer et al., 1993; Lanteri et al., 1995b; Yau et al., 1996) did, is a potentially important difference in anesthetic conditions. In our study, the lambs were anesthetized with isoflurane and not paralyzed, while in previous studies the individuals were only sedated (Bancalari et al., 1977; Yau et al., 1996) or anesthetized with a different anesthetic and paralyzed (Freezer et al., 1993; Lanteri et al., 1995b). Chloral hydrate and trichloroethylene, the sedative and anesthetic, respectively used in previous studies (Bancalari et al., 1977; Freezer et al., 1993; Lanteri et al., 1995b), do not affect airway caliber or R_rs_ (Colgan, 1965; Sandberg et al., 2013). On the other hand, isoflurane causes significant bronchodilation (Dikmen et al., 2003) and could have masked small differences in R_rs_ and R_L_ between shunt and control lambs.

To avoid the influence of the endotracheal tube resistance, the respiratory mechanics evaluation in the lambs of this study was performed using a calculated P_Tracheal_. The endotracheal tube is a major source of resistance and its inclusion on the evaluation of respiratory mechanics can add a significant noise, particularly to the resistance results (Sullivan et al., 1976; Fabry et al., 1994). Furthermore, the exclusion of P_resETT_ by calculating or directly measuring P_Tracheal_ can significantly improve the estimates of elastances and resistances (Lanteri et al., 1999; Karason et al., 2000) as well as the calculation of %E_2_ (Jandre et al., 2008). In the present study, P_Tracheal_ was calculated using previously published values of K_1_, K_2_, and inertance applied to the quadratic flow-dependent model (Guttmann et al., 2000). The direct measurement of P_Tracheal_ in the present study could have provided more accurate values of P_Tracheal_, but it would likely cause an excessive increase in P_resETT_ by the presence of a thin catheter through the small size ETTs used in the lambs of this study. The inclusion of the inertial term in the flow-dependent quadratic model used to calculate P_resETT_ in this study aimed to improve the accuracy of the calculated P_Tracheal_. This strategy significantly improved the accuracy of P_Tracheal_ calculation, achieving a maximal deviation between calculated and directly measured P_Tracheal_ to approximately 1 cmH_2_O (Guttmann et al., 2000). Lastly, the inclusion of the inertance term in the flow-dependent quadratic model of P_resETT_ significantly improved the accuracy of the assessment of tidal recruitment/derecruitment and overdistention measured by %E_2_ in a simulation study (Jandre et al., 2008).

The inclusion of the inertance term on the volume-dependent single compartment model used to estimate respiratory mechanics in this study aimed to account for the effects of inertial forces associated with the relatively high *f*_R_ used to maintain the target P_ET_CO_2_. Inertance has been shown to significantly contribute to this model at *f*_R_ higher than 20 bpm (Lanteri et al., 1999). In addition, E_rs_ and E_L_ were significantly underestimated with no effect on R_rs_ and R_L_ when inertance was omitted from the model (Lanteri et al., 1999). In a simulation study, Turner et al. (Turner et al., 1991) demonstrated that the overestimation of C_rs_, when the inertial term of the model was omitted, was more significant in conditions of higher C_rs_, as in the control lambs. As presented in Supplementary Table S3, the same results were observed in the lambs of this study when the inertance term was omitted. In the control and shunt lambs, C_rs_ and C_L_ were overestimated when the model excluding the inertial term was used. In addition, C_cw_ and R_cw_ were underestimated and R_rs_ was underestimated in control lambs when the model without inertance was applied.

### Gas exchange

The most important gas exchange abnormality reported in awake infants (Lees et al., 1967) and anesthetized children (Fletcher et al., 1986) with CHD and increased PBF is an impairment in arterial oxygenation resulting from increased pulmonary ventilation/perfusion (V̇/Q̇) mismatch (Fletcher, 1993). Differently, the shunt lambs of this study presented no significant difference in arterial oxygenation when compared to the control and SHAM animals. However, the small number of lambs used in this study likely precluded us to find significant difference in arterial oxygenation, particularly in P_(A-a)_O_2_. As previously reported in children (Fletcher et al., 1986), the physiologic dead space of the shunt individuals was not different than in the control ones. This finding was also observed in pigs where cardiac output was increased with dobutamine (Mosing et al., 2015). In addition, an increase in SIII has also been observed in children (Fletcher et al., 1986) and pigs (Mosing et al., 2015) with increased PBF. This finding was not observed in the shunt lambs even when the SIII was normalized to different sizes of V_T_. The increase in SIII observed when the pulmonary circulation is overloaded seems to occur due to an increased spread of V̇/Q̇ in the lungs (Fletcher et al., 1986; Fletcher, 1993). The lack of difference in SIII_n_ between shunt and control lambs could be related to the absence of left ventricular failure in the shunt lambs since the pulmonary gas exchange abnormalities in the presence of increased PBF are expected to be less in the absence of left ventricular failure (Fletcher, 1993). Moreover, the use of PEEP of 5 cmH_2_O after an alveolar recruitment maneuver in the lambs of this study could have minimized the V̇/Q̇ mismatch caused by the increased PBF, as observed when a similar ventilatory strategy was used in infants with CHD (Sun et al., 2020). Lastly, the prone position used during the experiments possibly contributed to minimize the pulmonary gas exchange derangements caused by the increase in PBF and PAp observed in this model of CHD, as observed in a swine model of ARDS (Katira et al., 2021).

## Limitations

The results of this study should be interpreted in the light of important limitations. The absence of functional residual capacity, lung aeration distribution, concurrent pulmonary to systemic blood flow and pulmonary artery pressure measurements, as well as the lack of direct measurement of P_Tracheal_ during the experiments were already discussed in previous paragraphs.

The small sample size used in this study may have underpowered the study to detect significant differences in some variables such as body weight and arterial oxygenation. Nevertheless, we were able to identify significant differences in important variables of pulmonary function such as C_rs_, C_L_, C_cw_ and R_cw_.

The prone position adopted in the lambs of this study aimed to reproduce the natural position of these animals in their awake state. It is important to highlight that prone position reduces the vertical gradient of pleural pressure maintaining a more homogeneous distribution of ventilation and consequently C_L_, as well as improve arterial oxygenation (Katira et al., 2021). Therefore, the results of this study should be carefully applied to other recumbencies, especially the supine position, which is the typical position used for sedated and mechanically ventilated human patients undergoing diagnostic procedures. Likely, the respiratory mechanics abnormalities identified in the shunt lambs of this study would be exacerbated in supine position, as previously observed in animal models of early lung injury (Katira et al., 2021).

Previously, we have demonstrated that SHAM fetal surgery did not contribute to the cardiovascular and pulmonary characteristics of shunt lambs (Reddy et al., 1995; Oishi et al., 2008). To evaluate any effect the thoracotomy itself might have subsequent on parameters of respiratory mechanics, we performed pulmonary function testing in SHAM lambs that had undergone a late gestation fetal thoracotomy without placement of an aortopulmonary graft. When compared with unoperated age-matched control lambs, we found no differences in any of the respiratory mechanics variables; therefore, we conclude that the lower C_rs_ and C_L_ in the shunt lambs were intrinsic to the pulmonary derangements resulting from the post-natal effects of the *in-utero* placement of the aortopulmonary graft. This conclusion is corroborated by previous studies reporting that C_rs_ and C_L_ improved after the correction of the pulmonary overcirculation in children with CHD (Naulty et al., 1978; Lanteri et al., 1995b). In addition, the normalization of compliances and resistances by the actual body weight of the lambs may have overestimated their values in the shunt lambs. Infants with CHD commonly fail to thrive with retarded growth and lower body mass index when compared to normal infants (Menon and Poskitt, 1985). To minimize the possible interference of this issue, previous studies normalized compliances to height (Lanteri et al., 1995b; Yau et al., 1996) or to functional residual capacity (Bancalari et al., 1977; Baraldi et al., 1993). No other morphometric measurements were available for the lambs used in this study. However, this limitation has minimal impact on our results and conclusions since there was no significant difference in body weight between groups. Paralysis was not provided in the lambs of our study mainly because of ethical concerns raised by our IACUC in regard to the monitoring of adequate depth of anesthesia during the experiments (Drummond et al., 1996). Because the use of neuromuscular blockers during passive mechanical ventilation does not seem to affect the respiratory mechanics in anesthetized individuals (Behrakis et al., 1983), we decided to not rebut our IACUC recommendation. Nonetheless, we closely monitored P_eso_, P_aw_ and V̇ during the experiments to guarantee that no activity of respiratory muscles affected our respiratory mechanics data.

The normal body temperature of lambs varies between 37.9 and 40.2°C (Hamdy and Weaver, 1958), which is higher than the default temperature of our blood gas machine (37°C). To avoid errors related to the human specific algorithm to correct the blood gas results for the actual temperature of the lambs, we decided to not use temperature-corrected values for our blood gas results and for the calculation of P_(A-a)_O_2_. In fact, most lambs presented negative P_(A-a)_O_2_ when temperature-corrected values of PaO_2_ we used.

## Conclusion

In shunt lambs, R_cw_, C_rs_ and C_L_ were decreased and C_cw_ was increased when compared to control lambs. The characteristics of the respiratory mechanics in this surgical ovine model of CHD are consistent with what has been described previously for infants and children with CHD and increased PBF and pressure.

## Data Availability

The original contributions presented in the study are included in the article/Supplementary Material, further inquiries can be directed to the corresponding authors.
