# Supplementary material for: Respiratory mechanics and gas exchange in an ovine model of congenital heart disease with increased pulmonary blood flow and pressure
Source: Front Physiol. 2023 Jun 9;14:1188824. doi: 10.3389/fphys.2023.1188824 (PMC10288580; doi:10.3389/fphys.2023.1188824)
Supplement: Supplementary file 1 [file DataSheet1.docx]

Supplementary Material

Respiratory mechanics in an ovine model of congenital heart disease with increased pulmonary blood flow and pressure.

**Joao H. N. Soares^1^, Gary W. Raff^2^, Jeffrey R. Fineman^3,4^, and Sanjeev A. Datar^4^**

**1 – Department of Surgical and Radiological Sciences, School of Veterinary Medicine, University of California, Davis.**

**2 – Department of Surgery, School of Medicine, University of California, Davis.**

**3 – Cardiovascular Research Institute, University of California, San Francisco.**

**4 – Department of Pediatrics, School of Medicine, University of California, San Francisco.**

*Corresponding author:

Joao H. N. Soares – [jhsoares@ucdavis.edu](mailto:jhsoares@ucdavis.edu)

Department of Surgical and Radiological Sciences, School of Veterinary Medicine, University of California, Davis. One Shields Avenue, Davis, CA, USA – 95616.

Phone (office): (530) 752-7398

Phone (cell phone): (530) 979-1367

*Co-corresponding author:

Sanjeev A. Datar – [sanjeev.datar@ucsf.edu](mailto:sanjeev.datar@ucsf.edu)

# Supplementary Data

**Esophageal Catheter Construction and Positioning:**

Esophageal pressure (P_eso_) was measured by a custom-made balloon catheter positioned in the esophagus. The catheter was made with a polyethylene tubing (240 – BD Intramedic, MD, USA) of 100 cm and a non-lubricated latex condom (Atlas, Global Protection Corp, MA, USA). Multiple lateral holes were performed in the entire 9 cm distal end of the PE tubing for adequate pressure transmission. A 16-gauge blunted needle was inserted to the proximal end of the catheter to connect it to a calibrated differential pressure transducer (P75, Hugo Sacks Elektronik-Harvard Apparatus, Germany). The balloon was made from the condom, which was cut at approximately 12 cm, placed at the distal end of the PE tubing covering all holes and tightly tied at the 10 cm distal end of the tubing with silk suture (0). The balloon catheter was checked for leaks by submerging it in water while pressurized at 30 cmH_2_O. If any leak was detected during the test, the balloon was removed and made from a new condom until no leak was detected.

The esophageal balloon catheter was positioned in the caudal part of the thoracic esophagus using a technique described by (1). First, the length from the incisors to the mid thoracic region was estimated from each animal’s topographic anatomy. Then, with the balloon completely deflated the catheter was orally introduced in the esophagus up to the pre-estimated length and connected to the pressure transducer. The catheter was initially filled with 10 mL and 5 mL were immediately withdrew to maintain 1 mL within the balloon. If a positive deflection was observed after a gentle compression of the left cranial abdomen, the catheter was pulled back until no positive deflection was observed. If too much interference from cardiac oscillations were observed in the P_eso_ signal displayed in a dedicated monitor, the catheter was moved slightly cranially or caudally in the esophagus. Subsequentially, an external compression of the thorax with the endotracheal tube occluded was performed (positive pressure occlusion test) in the absence of spontaneous effort while a real-time scatter plot with airway opening pressure (P_ao_) in the “y” axis and P_eso_ in the “x” axis was displayed on the monitor. The esophageal balloon catheter was considered properly positioned if the ratio between P_eso_ and P_ao_ variations (ΔP_eso_/ΔP_ao_) are within 0.9 to 1.1 and the slope of the linear regression between P_eso_ and P_ao_ were within 0.95 and 1.05 as shown in figure S1. If these criteria were not achieved at first, the balloon was inflated or deflated at 0.5 mL steps followed by small adjustments on its position in the esophagus until the criteria for proper positioning were met (2).


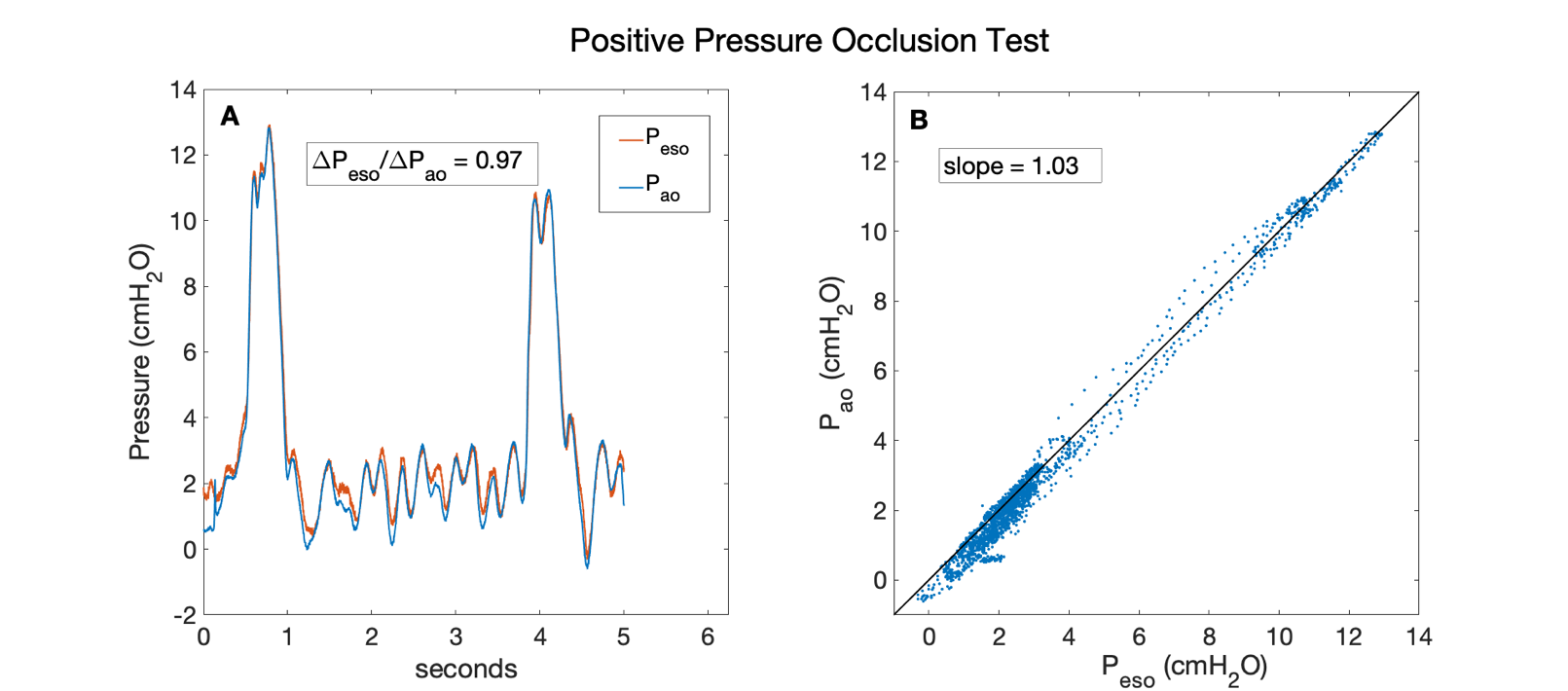
Figure S1. Esophageal (P_eso_) and airway opening (P_ao_) pressures graphs during the positive pressure occlusion test performed in one of the shunt lambs. Graph A: P_eso_ and P_ao_ scalars over time were simultaneously displayed during the positive pressure occlusion maneuver. The ratio between P_eso_ and P_ao_ variations (ΔP_eso_/ΔP_ao_) was 0.97, demonstrating the proper positioning of the esophageal balloon catheter. Graph B: The scatterplot of P_ao_ and P_eso_ was displayed in real-time during the positive pressure occlusion maneuver for the assessment of its slope. In this example, a slope of 1.03 was noted, also indicating the proper positioning of the catheter and the proper pleural pressure transmission to it.

**Respiratory Mechanics:**

The respiratory mechanics assessment on the lambs of this study was performed excluding the endotracheal tube resistive pressure (P_ResETT_), which was calculated for each lamb utilizing the quadratic flow-dependent model below:

P_ResETT_ (t) = (K_1ETT_ + K_2ETT_ × |V̇|(t)) × V̇ (t) + In_ETT_ × V̇̇; (1)

where t is time, V̇ was flow, V̇̇ the acceleration of the gases, and K_1ETT_, K_2ETT_ and In_ETT_ were the endotracheal tube values of linear resistance, flow-dependent resistance and inertance, respectively, obtained from reported values of ETT sizes 5.0 to 6.5 (3) and 7.0 (4). To exclude the resistive properties of the ETT on the estimates of respiratory system and lung mechanics, tracheal pressure (P_Tracheal_) was calculated for the entire respiratory cycle by P_ao_ (t) – P_ResETT_ (t). Peak and mean P_resETT_ achieved in all experiments were calculated from the absolute values of P_resETT_ and reported in Table S1. Figure S1 presents the calculated P_resETT_ and P_ao_ with and without P_resETT_ in one of the shunt lambs.

Dynamic respiratory mechanics was evaluated using a single-compartment model including a volume-dependent elastance and an inertial pressure term. Respiratory system, lung and chest wall respective volume-independent elastance (E_1rs_, E_1L_, and E_1cw_), volume-dependent elastance (E_2rs_, E_2L_, and E_2cw_), resistance (R_rs_, R_L_, and R_cw_) and inertance (In_rs_, In_L_, and In_cw_) were estimated by multiple linear regression (least square method) of the following models of the equation of motion using their respective driving pressures (5).

P_Tracheal_ (t) = In_rs_ × V̇̇ + R_rs_ × V̇(t) + (E_1rs_ + E_2rs_ × V(t)) × V(t)+ P_Tracheal,0_ (2)

P_tp_(t) = In_L_ × V̇̇ + R_L_ × V̇(t) + (E_1L_ + E_2L_ × V(t)) × V(t) + P_tp,0_ (3)

P_eso_(t) = In_cw_ × V̇̇ + R_cw_ × V̇(t) + (E_1cw_ + E_2cw_ × V(t)) × V(t) + P_eso,0_ (4)

where V is volume, P_tp_ is transpulmonary pressure (P_Tracheal_ – P_eso_), and P_Tracheal,0_, P_tp,0_, and P_eso,0_ are P_Tracheal_, P_tp_ and P_eso_, respectively, when V, V̇ and V̇̇ are zero. Compliance was calculated for the total respiratory system (C_rs_), chest wall (C_cw_) and lungs (C_L_) by 1/E_rs_, 1/E_cw_ and 1/E_L_, respectively, and normalized to the body weight in kg of each lamb. R_rs_, R_L_ and R_cw_ were normalized to the body weight of each lamb using a power law described by Stahl (6). All estimates of elastances, resistances and inertances were reported as their average during the last 30 cycles of ventilation before the arterial blood sampling. Total elastance of respiratory system (E_rs_), lung (E_L_) and chest wall (E_cw_) were calculated by the sum of their respective volume-dependent and independent terms (total elastance = E_1_ + E_2_ × V_T_). The parameters estimated from models 2, 3 and 4 are presented on Table S2.

An inertial term (In × V̇̇) to describe pressure changes associated with the acceleration of gases was included in the models to improve the accuracy of elastance and resistance estimates since a relatively high *f*_R_ (20 to 40 breaths/minute) was used during the experiments (7). Table S3 presents the comparison of compliances and resistances estimated by the models including or not the inertial pressure term. In both groups of lambs, C_rs_ and C_L_ were overestimated when the inertial term was omitted.

|  | All  (n=20) | ETT 5.0  (n = 3) | ETT 5.5 (n=12) | ETT 6.0  (n=4) | ETT 6.5  (n=1) |
| --- | --- | --- | --- | --- | --- |
| Peak P_resETT_  (cmH_2_O) | 3.5 (2.0 4.8) | 4.0 (3.1 4.8) | 2.0 (3.2 4.6) | 3.5 (2.4 3.9) | 3.3 |
| Mean P_resETT_  (cmH_2_O) | 0.9 (0.7 2.0) | 0.9 (0.8 1.2) | 0.9 (0.7 2.0) | 1.1 (0.8 1.3) | 0.8 |

Table S1. Mean and peak endotracheal tube resistive pressure (P_resETT_) calculated for each endotracheal tube used in this study. Data from all animals used in this study (7 control, 7 SHAM, and 6 shunt lambs.) are presented altogether or separated by endotracheal tube (ETT) sizes. Values are expressed as median (range).


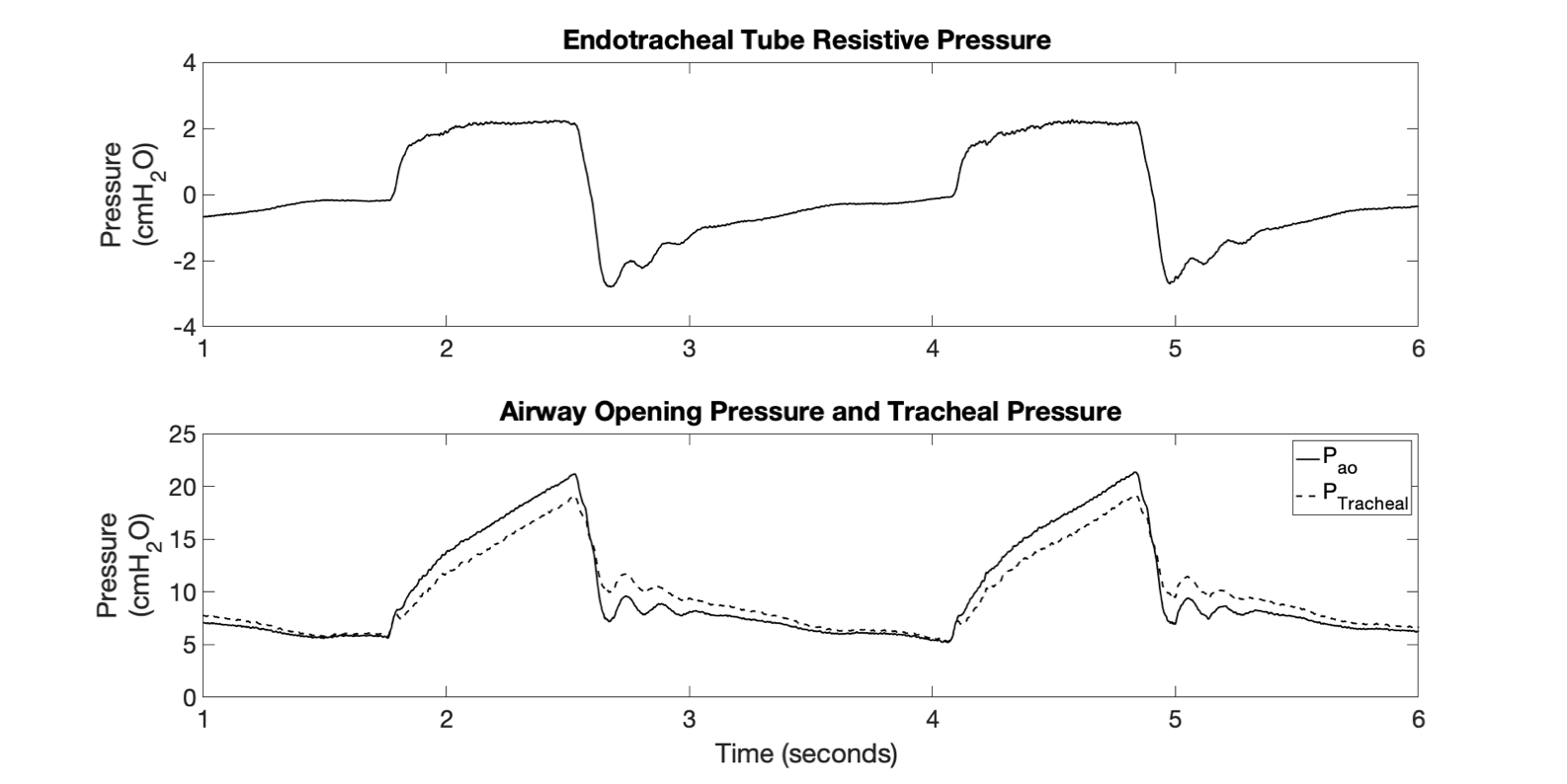
Figure S1. Measured airway opening pressure (P_ao_) and calculated endotracheal tube resistive pressure (P_resETT_ – Equation 1) and tracheal pressure (P_Tracheal_ - calculated as P_ao_ – P_resETT_) in one representative shunt lamb.

Table S2. Estimated parameters and the coefficient of determination (R^2^) of the volume-dependent single compartment model used to evaluate respiratory mechanics in the control, SHAM, and shunt lambs of this study. Values are expressed as median (range). Tracheal pressure (P_Tracheal_), transpulmonary pressure (P_tp_) and esophageal pressure (P_eso_) were the driving pressure used in the models for the respiratory system, lung and chest wall mechanics evaluation, respectively. R is resistance, E_1_ is the volume-independent elastance, E_2_ is the volume-dependent elastance, In is inertance, P_0_ is the driving pressure of each model when volume, flow and acceleration are 0.

|  | Driving Pressure | R | E_1_ | E_2_ | In | P_0_ | R^2^ |
| --- | --- | --- | --- | --- | --- | --- | --- |
| CONTROL | P_Tracheal_ | 5.4  (2.2 11) | 51  (38 64) | 35  (-36 125) | 33  (16 70) | 5.5  (5.4 5.9) | 0.9953  (0.9844 0.9976) |
|  | P_tp_ | 3.7  (1.0 9.8) | 21  (18 29) | 34  (-45 99) | 36  (24 72) | 2.5  (1.2 4.2) | 0.9691  (0.9427 0.9814) |
|  | P_eso_ | 1.7  (1.0 2.2) | 32  (22 38) | 13  (-9.4 33) | -5.1  (-8.8 -2.2) | 3.1  (1.5 4.7) | 0.9791  (0.9428 0.9936) |
| SHAM | P_Tracheal_ | 7.7  (2.2 13) | 54  (39 80) | -3.6  (-58 55) | 49  (-98 67) | 5.5  (5.4 7.1) | 0.9922  (0.9767 0.9950) |
|  | P_tp_ | 6.1  (1.3 12) | 24  (14 45) | -42  (-104 3.0) | 63  (87 75) | 0.8 (0.0 2.2) | 0.9649  (0.8973 0.9807) |
|  | P_eso_ | 1.6  (0.9 2.3) | 26  (22 43) | 26  (9.6 97) | -10  (-22 -5.9) | 4.8  (4.5 5.7) | 0.9927  (0.9221 0.9975) |
| SHUNT | P_Tracheal_ | 8.3  (0.5 14) | 89  (61 112) | 100  (49 371) | 36  (4.2 48) | 5.6  (4.9 5.8) | 0.9893  (0.9861 0.9958) |
|  | P_tp_ | 7.4  (0.6 14) | 58  (37 79) | 122  (31 358) | 34  (8.4 45) | 4.2  (1.4 5.7) | 0.9825  (0.9764 0.9927) |
|  | P_eso_ | 0.8  (0.4 1.2) | 27  (14 38) | -2.3  (-51 17) | 0.8  (-3.8 4.2) | 1.5  (-0.8 4.2) | 0.9754  (0.9601 0.9911) |

Table S3: Respiratory system, lungs, and chest wall compliances (C_rs_, C_L_, and C_cw_) and resistances (R_rs_, R_L_, and R_cw_) in control and shunt lambs estimated from a volume-dependent single compartment model of the equation of motion including (VDSCM_In_) or not (VDSCM) the inertial pressure term. Values are expressed as mean ± 95% confidence interval. * = significant difference between values of VDSCM and VDSCM_In_

|  | Model | | C_rs_  (mL/kg/cmH_2_O) | C_L_  (mL/kg/cmH_2_O) | C_cw_  (mL/kg/cmH_2_O) | R_rs_  (cmH_2_O/L/s/kg^-0.7^) | R_L_  (cmH_2_O/L/s/kg^-0.7^) | R_cw_  (cmH_2_O/L/s/kg^-0.7^) |
| --- | --- | --- | --- | --- | --- | --- | --- | --- |
| Control (n=7) | | VDSCM_In_ | 1.32 ± 0.19 | 3.06 ± 0.69 | 2.37 ± 0.36 | 39.3 ± 23.6 | 29.3 ± 24.1 | 9.98 ± 1.16 |
|  |  | VDSCM | 1.39 ± 0.20 | 3.53 ± 0.87 | 2.34 ± 0.35 | 39.4 ± 23.6 | 29.3 ± 24.1 | 9.97 ± 1.16 |
|  |  | *P* value | 0.0005* | 0.0021* | 0.0039* | 0.0082* | 0.068 | 0.0457* |
| Shunt  (n=6) | | VDSCM_In_ | 0.93 ± 0.23 | 1.27 ± 0.31 | 3.57 ± 1.18 | 40.8 ± 23.7 | 36.1 ± 24.6 | 4.72 ± 1.79 |
|  |  | VDSCM | 0.96 ± 0.25 | 1.34 ± 0.36 | 3.58 ± 1.22 | 40.8 ± 23.7 | 36.1 ± 24.6 | 4.72 ± 1.79 |
|  |  | *P* value | 0.0473* | 0.0210* | 0.4499 | 0.1314 | 0.4118 | 0.3354 |

**Pulmonary to systemic blood flow ratio and pulmonary artery pressure data:**

Pulmonary to systemic blood flow ratio (Q̇_p_/Q̇_s_) and mean pulmonary artery pressure (MPAP) were subsequently obtained in 5 of the shunt lambs used in this study at a later phase of this project (2 to 10 weeks later). The lambs were anesthetized using a similar anesthetic protocol as the one used for the present study. A pulmonary artery catheter was percutaneously placed and advanced into the pulmonary artery, and its correct position was verified by fluoroscopy and the typical blood pressure waveforms displayed on the monitor during catheterization. MPAP was measured by a calibrated pressure transducer (Philips Healthcare, Cambridge, MA). Q̇_p_/Q̇_s_ was calculated using the Fick principle as previously described (8). Table S4 presents the average values of MPAP and Q̇_p_/Q̇_s_ obtained in 5 shunt lambs.

Table S4: Pulmonary to systemic blood flow ratio (Q̇_p_/Q̇_s_) and mean pulmonary artery pressure (MPAP) measured in 5 shunt lambs investigated aging between 8 and 15 weeks.

| Lamb | Q̇_p_/Q̇_s_ | MPAP (mmHg) |
| --- | --- | --- |
| 1 | 2.3 | 26 |
| 2 | 2.5 | 33 |
| 3 | 2.2 | 45 |
| 4 | 2.0 | 30 |
| 5 | 2.6 | - |
| Mean ± SD | 2.3 ± 0.2 | 34 ± 8 |

**References:**

1. **Lanteri CJ, Kano S, and Sly PD**. Validation of esophageal pressure occlusion test after paralysis. *Pediatr Pulmonol* 17: 56-62, 1994.

2. **Mojoli F, Torriglia F, Orlando A, Bianchi I, Arisi E, and Pozzi M**. Technical aspects of bedside respiratory monitoring of transpulmonary pressure. *Ann Transl Med* 6: 377, 2018.

3. **Guttmann J, Kessler V, Mols G, Hentschel R, Haberthur C, and Geiger K**. Continuous calculation of intratracheal pressure in the presence of pediatric endotracheal tubes. *Crit Care Med* 28: 1018-1026, 2000.

4. **Sullivan M, Paliotta J, and Saklad M**. Endotracheal tube as a factor in measurement of respiratory mechanics. *J Appl Physiol* 41: 590-592, 1976.

5. **Lanteri CJ, Kano S, Nicolai T, and Sly PD**. Measurement of dynamic respiratory mechanics in neonatal and pediatric intensive care: the multiple linear regression technique. *Pediatr Pulmonol* 19: 29-45, 1995.

6. **Stahl WR**. Scaling of respiratory variables in mammals. *J Appl Physiol* 22: 453-460, 1967.

7. **Lanteri CJ, Petak F, Gurrin L, and Sly PD**. Influence of inertance on respiratory mechanics measurements in mechanically ventilated puppies. *Pediatr Pulmonol* 28: 130-138, 1999.

8. **Reddy VM, Meyrick B, Wong J, Khoor A, Liddicoat JR, Hanley FL, and Fineman JR**. In utero placement of aortopulmonary shunts. A model of postnatal pulmonary hypertension with increased pulmonary blood flow in lambs. *Circulation* 92: 606-613, 1995.
